# Performance Evaluation of GPS Auto-Surveying Techniques

**DOI:** 10.3390/s21217374

**Published:** 2021-11-06

**Authors:** João Manito, José Sanguino

**Affiliations:** Instituto de Telecomunicações, Instituto Superior Técnico, Av. Rovisco Pais, 1049-001 Lisboa, Portugal; sanguino@lx.it.pt

**Keywords:** GNSS, GPS, base-station, auto-surveying

## Abstract

With the increase in the widespread use of Global Navigation Satellite Systems (GNSS), increasing numbers of applications require precise position data. Of all the GNSS positioning methods, the most precise are those that are based in differential systems, such as Differential GNSS (DGNSS) and Real-Time Kinematics (RTK). However, for absolute positioning, the precision of these methods is tied to their reference position estimates. With the goal of quickly auto-surveying the position of a base station receiver, four positioning methods are analyzed and compared, namely Least Squares (LS), Weighted Least Squares (WLS), Extended Kalman Filter (EKF) and Unscented Kalman Filter (UKF), using only pseudorange measurements, as well as the Hatch Filter and position thresholding. The research results show that the EKF and UKF present much better mean errors than LS and WLS, with an attained precision below 1 m after about 4 h of auto-surveying. The methods that presented the best results are then tested against existing implementations, showing them to be very competitive, especially considering the differences between the used receivers. Finally, these results are used in a DGNSS test, which verifies a significant improvement in the position estimate as the base station position estimate improves.

## 1. Introduction

The Global Navigation Satellite System (GNSS) has, since its introduction in 1978, revolutionized several industries, such as aviation, transportation and agriculture, with a continued increase in the number of GNSS-enabled devices for the foreseeable future. Most of these devices make use of the positioning capabilities provided by GNSS and show that there is a strong and dynamic market for precise positioning capabilities [1].

A single-frequency GNSS receiver can estimate its position with an accuracy of less than 10 m. To improve these results, high-quality dual-frequency GNSS receivers can attain position solutions with an error below 1 or 2 m [2]. These receivers are usually very expensive, on the order of thousands of euros, although newer low-cost receivers have recently appeared on the market [3].

While these receivers can be enough for many applications, others require an accuracy down to the decimeter or centimeter level [3,4]. To solve this problem, a GNSS receiver can employ differential methods such as Differential GNSS (DGNSS) and Real-Time Kinematics (RTK), which, for absolute positioning, make use of a static reference receiver, a base station, with a known position. DGNSS positioning, in its more usual form, is based on comparing the pseudorange obtained by the reference station to a given satellite with the expected pseudorange given the same satellite and the previously surveyed reference station position. Given the spatial correlation of the GNSS signals, the pseudorange error of the reference station to a given satellite will be similar to that of another receiver nearby, and as such a correction factor can be obtained and applied to the received pseudoranges of the rover receiver. In RTK, the process is similar, but instead of using only pseudoranges, the positioning is based on the measurement of both the pseudorange and carrier phase of the received signals. A reference station with a known position creates a set of carrier phase measurements that can then be transmitted to a rover receiver, which in turn will create a Double Difference solution and solve the the integer ambiguity problem of the carrier phase observable, allowing for much more precise results than those possible with simply pseudoranges [2]. It should be noted, however, that this only applies to RTK usage for absolute positioning. In the case of relative positioning using RTK, as with attitude estimation, the position estimate of the base station is unnecessary; however, that application is outside of the scope of the present paper.

**Figure 1:**
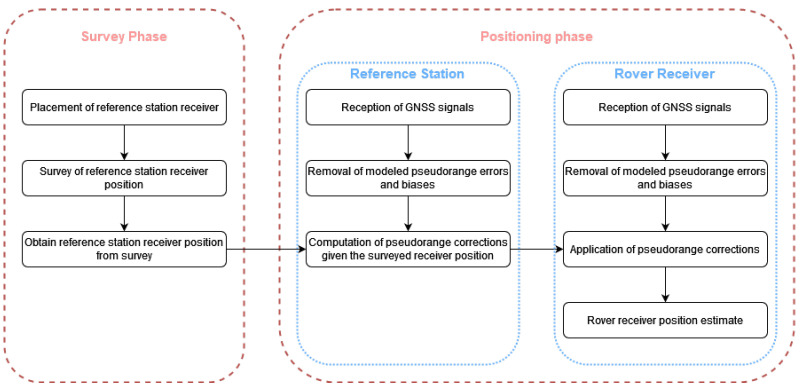
Overview of the DGNSS positioning method.

**Figure 2:**
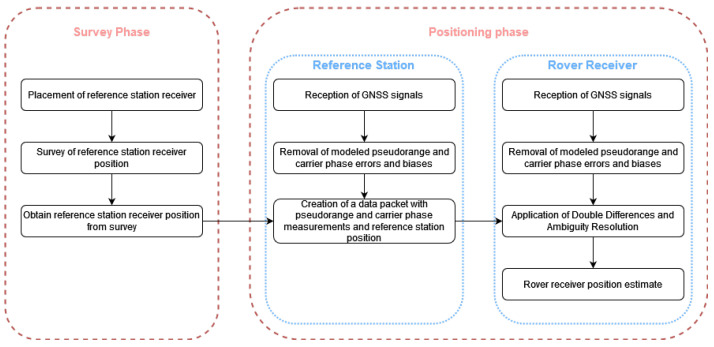
Overview of the RTK positioning method.

The problem this paper intends to tackle is how to obtain a precise estimate for the position of a base station, a so-called auto-survey, which then can be used to obtain the necessary corrections for a differential positioning system. This problem is part of the “Survey Phase” illustrated in Figure 1 and Figure 2 and a critical part of the entire differential positioning system. Current research trends in the GNSS precise static positioning area are focused on the application of Precise Point Positioning (PPP) [5,6,7] for the problem of static receiver positioning. If a base station already exists, both RTK [3,8,9] and DGPS [10] can also be used for determining the position of a new base station. However, these methods require external communications, usually in the form of internet access or radio links as well as at least one already existing base station, making them unsuitable for remote applications and/or for deployment in developing countries. In an attempt to solve this problem, this paper aims to characterize position estimation methods that require no external connectivity and can be used in low-cost setups, with regards to both the precision and time of convergence of the position solution. While high-end receivers can output not only pseudorange and carrier phases but also Doppler and signal-to-noise ratios, for example, the research presented in this paper was restricted to those observables that are guaranteed to be present in low-end receivers. For the position estimation, four positioning methods were chosen: Least Squares (LS), Weighted Least Squares (WLS), Extended Kalman Filter (EKF) and Unscented Kalman Filter (UKF). The first two are based on the Least Squares method of data fitting and attempt to obtain the best position solution possible for a set of observations in a given instant, with no regards for previous observations. The other two methods are based on the theory of the Kalman Filter and provide a way to incorporate previous measurements in the current position estimate, as well as including extra information regarding the receiver’s dynamics. Besides these methods, a code-smoothing algorithm, the Hatch Filter, was also implemented in order to use carrier phase measurements to improve the pseudorange measurements. Finally, a position threshold filter based on the standard deviation of the estimates was implemented.

The novelty of this paper is that it not only attempts to fill the gap of a complete comparison between these methods using modern low-cost receivers, it also intends to demonstrate the viability of these methods for DGNSS/RTK base station auto-survey using only the measurements available with low-cost single-frequency receivers capable of raw data output By characterizing and benchmarking the time and precision obtained using short-term surveys for the base station position in a completely autonomous fashion, it aims to provide information regarding the trade-off between the survey time and precision of the position estimate, as well as analyzing its impact on the positioning accuracy of a rover receiver. The low-cost, fully-autonomous approach has applicability in several areas where the required position estimate precision is greater than what is obtainable with a single, low-cost receiver but cannot justify the added expense of a high-performance receiver, as well as temporary applications that, by their nature, require a quick survey period for the base station. Such applications can, for example, include the following:

- Non-professional or associative sporting events, such as orienteering or nautical sports;
- Forestry applications that do not necessitate permanent base stations for monitoring and registry activities;
- Use of drones in temporary or remote activities such as power line monitoring or the monitoring of forest or crop areas;
- Agricultural applications for more precise crop dusting and monitoring using airplanes and helicopters, where the the expense of a full precision-agriculture suite of machinery is prohibitive for the owners;
- Other temporary activities that are performed in correction-denied areas that do not require centimetric precision.

While PPP provides better precision for the problem of single-receiver positioning, it also requires access to external data that must be obtained either a priori or during the survey; in comparison, our methodology is totally autonomous, and this paper aims to show it is a viable alternative. In addition, the proposed approach allows the usage of very low-cost processors for the positioning algorithm, since the pseudorange-only approach is computationally much simpler due to not having to solve the integer ambiguity problem, as well as including computationally simple smoothing and filtering methods. As a result, the proposed methodology leads the way to lower entry costs for precise position estimation in remote areas with no connectivity or where the required communication systems are expensive. It is worthy of note that, while this paper worked only with GPS measurements, its methodology can be easily applied to other GNSS systems, which should improve the position estimate accuracy [11,12].

## 2. Materials and Methods

### 2.1. GPS Observables

In this paper, the only GPS observables used are the pseudorange and carrier phase as given by a GPS receiver capable of raw data output.

#### 2.1.1. Pseudorange

For a satellite–receiver pair, let $r^{i}$ be the true range from the receiver to the *i-th* satellite. This range can be calculated from the transit time of the GPS signal; however, both receiver and satellite present some errors in their clocks, $\delta t_{r}$ and $\delta t^{i}$, respectively, which means that the transit time calculation yields a range different from the true range. Other errors, such as the tropospheric and ionospheric delays, $T^{i}$ and $I^{i}$, respectively, and multipath errors, $MP^{i}$, also add to this difference. This new distance is called the pseudorange and is given by [13]

(1) $$\rho^{i}=R^{i}+c\delta t_{r}-c\delta t^{i}+T^{i}+I^{i}+MP^{i}+\epsilon_{\rho }$$

where all terms are in units of meters and $\epsilon_{\rho }$ corresponds to other unmodeled errors. The true range $R^{i}$ can be given by [2]:

(2) $$R^{i}=\sqrt{{\left(x^{i}-x\right)}^{2}+{\left(y^{i}-y\right)}^{2}+{\left(z^{i}-z\right)}^{2}}$$

where (x,y,z) are the receiver coordinates, and $\left(x^{i},y^{i},z^{i}\right)$ are the *i-th* satellite coordinates.

#### 2.1.2. Carrier Phase

Another observable is the phase of the GPS carrier signal, which can be measured with a resolution on the order of millimeters but is ambiguous by an integer number of cycles, also called integer ambiguity, $N_{r}^{s}$. By multiplying the carrier phase by the wavelength of the GPS signal and adding the same errors as the pseudorange model, the full model of the carrier phase observable, in units of meters, can be written as [13]

(3) $$\Phi_{r}^{s}=R+c\delta t_{r}+c\delta t^{s}+\lambda N_{r}^{s}+T^{i}-I^{i}+MP^{i}+\epsilon_{\phi }$$

where $\Phi_{r}^{s}$ is the carrier phase observable in meters, the modeled errors are equal to those in Equation (1), and $\epsilon_{\phi }$ denotes the unmodeled errors in the carrier phase.

### 2.2. Position Determination

#### 2.2.1. Least Squares

Combining the pseudorange model of Equation (1) with Equation (2) and assuming the modeled errors have been removed from the pseudorange measurement, the pseudorange model can be rewritten as [2]

(4) $$\rho^{i}=\sqrt{{\left(x^{i}-x\right)}^{2}+{\left(y^{i}-y\right)}^{2}+{\left(z^{i}-z\right)}^{2}}+c\delta t_{r}$$

which is an equation with four unknowns: the receiver coordinates in an ECEF frame, $\left(x,y,z\right)$, and the receiver clock offset, $\delta t_{r}$. In order to solve this equation, at least four independent measurements are required, after linearizing the equations.

Since Equation (4) is non-linear, Taylor’s series expansion can be applied to the satellite–receiver range, with the linearization being conducted around an initial receiver position estimate $x_{0}=\left(x_{0},y_{0},z_{0}\right)$ [14]. By applying this linearization to Equation (4), it yields [14]

(5) $$\rho^{i}-R_{0}^{i}=\frac{x_{0}-x^{i}}{R_{0}^{i}}\Delta x+\frac{y_{0}-y^{i}}{R_{0}^{i}}\Delta y+\frac{z_{0}-z^{i}}{R_{0}^{i}}\Delta z+c\Delta t$$

where $R_{0}$ is the satellite–receiver distance for the initial position estimate. Since the problem requires at least four measurements, the previous equation can be rewritten in matrix form

(6) $$\begin{array}{cc} \; & \left[\begin{array}{c} \rho^{1}-R_{0}^{1}\\ \vdots \\ \rho^{n}-R_{0}^{n} \end{array}\right]=\left[\begin{array}{cccc} \frac{x_{0}-x^{1}}{R_{0}^{1}} & \frac{y_{0}-y^{1}}{R_{0}^{1}} & \frac{z_{0}-z^{1}}{R_{0}^{1}} & 1\\ \vdots & \vdots & \vdots & \vdots \\ \frac{x_{0}-x^{n}}{R_{0}^{n}} & \frac{y_{0}-y^{n}}{R_{0}^{n}} & \frac{z_{0}-z^{n}}{R_{0}^{n}} & 1 \end{array}\right]\left[\begin{array}{c} \Delta x\\ \Delta y\\ \Delta z\\ c\delta t \end{array}\right] \end{array}$$

or, in a more compact notation,

(7) Δρ=HΔx

Finally, the Least Squares solution of this equation is

(8) $$\Delta \hat{x}={\left(H^{\;T}H\right)}^{-1}H^{\;T}\Delta \rho$$

#### 2.2.2. Weighted Least Squares

The Least Squares solution of Equation (8) is taken with the assumption that the errors of the pseudorange measurements from all satellites have the same variance, which might not correspond to reality. To solve this, a weighting matrix, corresponding to the inverse of the measurement error covariance matrix, can be applied to the Least Squares solution [15].

While there are several possible formulations for the measurement error covariance, in this paper, the approach used in [16] is adopted. Let Q be the weighting matrix. Assuming uncorrelated measurements with a standard deviation given by

(9) $$\sigma_{i}=\frac{\sigma_{URA_{i}}}{\sin\epsilon_{i}}$$

where $\sigma_{URA_{i}}$ is the broadcasted user range accuracy for satellite *i* and $\epsilon_{i}$ is the satellite elevation angle, the weighting matrix can be constructed as a diagonal matrix with elements

(10) $$Q_{ii}=\frac{1}{\sigma_{i}^{2}}=\frac{\sin^{2}\epsilon_{i}}{\sigma_{URA_{i}}^{2}}$$

Using this matrix, the Weighted Least Squares solution can be written as [15]:

(11) $$\Delta \hat{x}={\left(H^{\;T}QH\right)}^{-1}H^{\;T}Q\Delta \rho$$

### 2.3. Extended Kalman Filter

The Extended Kalman Filter (EKF) is another possible method to obtain a position estimate. This method works in discrete time, $t_{k}$, and for each iteration *k* of the filter, there are two steps:

- Prediction, where the state vector ${\hat{x}}_{k}$ is estimated using the observations from the previous iteration;
- Filtering, where ${\hat{x}}_{k}$ is estimated using the state vector estimate of the prediction step and the current observations.

For the EKF, two different models are required: A dynamics model, which describes the receiver dynamics, and an observations model, which describes the relationship between the receiver state vector and the observations.

#### 2.3.1. Dynamics Model

There are several possible models for the receiver dynamics. In this paper, the P model for the EKF was implemented, as the reference receiver whose position is to be estimated is in a static, fixed position. In this model, the state vector x, state transition matrix Φ, and the noise covariance matrix Q are given by [17]

(12) $$x=\left[\begin{array}{c} x\\ y\\ z\\ x_{\phi }\\ x_{f} \end{array}\right]$$

(13) $$\Phi =\left[\begin{array}{ccccc} 1 & 0 & 0 & 0 & 0\\ 0 & 1 & 0 & 0 & 0\\ 0 & 0 & 1 & 0 & 0\\ 0 & 0 & 0 & 1 & \Delta t\\ 0 & 0 & 0 & 0 & 1 \end{array}\right]$$

(14) $$Q=\left[\begin{array}{ccccc} 0 & 0 & 0 & 0 & 0\\ 0 & 0 & 0 & 0 & 0\\ 0 & 0 & 0 & 0 & 0\\ 0 & 0 & 0 & q_{\phi } & 0\\ 0 & 0 & 0 & 0 & q_{f} \end{array}\right]$$

where $q_{\phi }$ and $q_{f}$ are associated with the Allan variance parameters. In the case of a low-cost temperature-compensated crystal oscillator, those parameters are approximated by [17]

(15) $$\begin{array}{cc} q_{\phi } & \approx \frac{h_{0}}{2}=\frac{2\cdot 10^{-19}}{2}\\ q_{f} & \approx 2\pi^{2}h_{-2}=2\pi^{2}\left(2\cdot 10^{-20}\right) \end{array}$$

The noise covariance matrix of the discrete-time dynamics model, $Q_{k}$, can be obtained from Equations (13) and (14):

(16) $$\begin{array}{cc} \; & Q_{k}\approx \Phi Q\Phi^{\;T}\Delta t\\ & =\Delta t\left[\begin{array}{ccccc} 0 & 0 & 0 & 0 & 0\\ 0 & 0 & 0 & 0 & 0\\ 0 & 0 & 0 & 0 & 0\\ 0 & 0 & 0 & c^{2}\left(q_{\phi }+\frac{q_{f}\Delta t^{2}}{3}\right) & \frac{c^{2}q_{f}\Delta t}{2}\\ 0 & 0 & 0 & \frac{c^{2}q_{f}\Delta t}{2} & c^{2}q_{f} \end{array}\right] \end{array}$$

with the clock variances multiplied by $c^{2}$ to convert the clock errors to units of meters.

#### 2.3.2. Observations Model

In the EKF, the non-linear navigation equations are linearized in order to construct the observations model. The observations equation is given by

(17) $$z_{k}=h\left[x(t_{k})\right]+v_{k}$$

where $z_{k}$ is the measured pseudorange vector with n≥4 observations, $v_{k}$ the observation noise and $h\left[x(t_{k})\right]$ is the navigation equation vector composed of *n* instances of Equation (4). The navigation equations can be linearized by obtaining the Jacobian matrix of $h\left[x(t_{k})\right]$, which yields the observation matrix, H. For the case of *n* observations, this matrix yields

(18) $$H_{k}=\left[\begin{array}{ccccc} \frac{x^{1}-\hat{x}}{{\hat{R}}^{1}} & \frac{y^{1}-\hat{y}}{{\hat{R}}^{1}} & \frac{z^{1}-\hat{z}}{{\hat{R}}^{1}} & 1 & 0\\ \vdots & \vdots & \vdots & \vdots & \vdots \\ \frac{x^{n}-\hat{x}}{{\hat{R}}^{n}} & \frac{y^{n}-\hat{y}}{{\hat{R}}^{n}} & \frac{z^{n}-\hat{z}}{{\hat{R}}^{n}} & 1 & 0 \end{array}\right]$$

where ${\hat{R}}^{i}$ is the satellite–receiver range, taken at the estimated position, given by Equation (2)

The last parameter of the observations model is the observation noise covariance matrix, $R_{k}$. In this paper, since the measurements are considered uncorrelated, the noise covariance matrix is reduced to a diagonal matrix, given by

(19) $$R_{k}=\left[\begin{array}{cccc} \sigma_{1}^{2} & \; & 0 & \\ & \sigma_{2}^{2} & \; & \\ & \; & \ddots & \\ & 0 & \; & \sigma_{n}^{2} \end{array}\right]$$

### 2.4. Unscented Kalman Filter

The Unscented Kalman Filter (UKF) is another implementation of the Kalman Filter for non-linear systems. However, instead of linearizing the non-linear system around a point, it computes the statistics of the state random variables, using a function called the Unscented Transform (UT), in order to approximate the state distribution of the non-linear function [18]. As such, the only difference between the EKF and UKF liess in its observations model, keeping the entire dynamics model from the EKF.

Let $f(x_{k})$ be a non-linear function, where x is a vector of Gaussian random variables with an expected value; i.e., mean, $\hat{x_{k}}$ and covariance matrix $P_{k}$. The UT of this function consists in taking a set of samples from the random variable $x_{k}$, applying the non-linear function to this set of points, and then obtaining a new set of statistics for the transformed random variable, which will also be Gaussian.

The points used in the UT are called sigma points and together form the sigma vector, X. For a given iteration *k*, these points are given by [18]

(20) $$\begin{array}{cc} X_{0,k} & ={\hat{x}}_{k}\\ X_{i,k} & ={\hat{x}}_{k}+\sqrt{n+\tau }{\left(\sqrt{P_{k}}\right)}_{i}\\ X_{i+n,k} & ={\hat{x}}_{k}-\sqrt{n+\tau }{\left(\sqrt{P_{k}}\right)}_{i} \end{array}\;\mathrm{with}\;i=1,\cdots ,n$$

where *n* is the size of x, τ is the scale factor of the sampling, ${\left(\sqrt{P_{k}}\right)}_{i}$ designates the *i*-th line of $\sqrt{P_{k}}$ and $\sqrt{P_{k}}$ can be obtained by the Cholesky decomposition of $P_{k}$. The resulting sigma vector is then a matrix of n×(2n+1), where each line is a set of sigma points for a given element of $x_{k}$. The scaling factor τ is used to adjust the spacing between sample points and their weight in the statistics of the transformation and can be either positive or negative. For the symmetrical sampling case [18],

(21) n+τ=3

After the sampling, the sigma points are propagated through f

(22) $$Y_{i},k=f\left(X_{i,k}\right)\;\mathrm{with}\;i=0,\cdots ,2n$$

and then the statistics of the resulting vector can be obtained by means of a weighted average [18], from which the mean and covariance matrix can be determined using

(23) $$\begin{array}{cc} \hat{y_{k}} & =\sum_{i=0}^{2n}W_{i}X_{i,k}\\ P_{yy} & =\sum_{i=0}^{2n}W_{i}\left(Y_{i,k}-\hat{y_{k}}\right){\left(Y_{i,k}-\hat{y_{k}}\right)}^{\;T} \end{array}$$

where the weight values $W_{i}$ are given by

(24) $$\begin{array}{cc} W_{0} & =\frac{\tau }{n+\tau }\\ W_{i} & =\frac{1}{2\left(n+\tau \right)} \end{array}$$

### 2.5. Hatch Filter

While the pseudorange measurements are unambiguous but imprecise, the carrier phase measurement is very precise but ambiguous. One method to apply carrier phase measurements without explicitly solving the integer ambiguity problem is to use those measurements to smooth the pseudorange measurements—so-called Carrier-Smoothed Code (CSC) techniques. One of these techniques is the Hatch Filter.

The time-differencing of the carrier phase measurement, in its error-free model, is given by [19]

(25) $$\Phi_{r}^{s}\left(n\right)-\Phi_{r}^{s}\left(n-1\right)=r_{n}-r_{n-1}+\lambda N_{r}^{s}-\lambda N_{r}^{s}=r_{n}-r_{n-1}$$

where, by means of differencing, the integer ambiguity is cancelled out. Then, the weighting factors W(n) for the Hatch Filter are given by

(26) W(n)=W(n−1)−γ

The weighting factor must be initialized in its first iteration, which can be accomplished by using the pseudorange measurement in this iteration, corresponding to making W(1)=1. Finally, the Hatch Filter equation is

(27) $$\begin{array}{cc} \rho_{s,k} & =W\left(n\right)\rho_{k}+\left(1-W\left(n\right)\right)\left(\rho_{s,k-1}+\Phi_{k}-\Phi_{k-1}\right) \end{array}$$

where γ is the averaging constant and defines the averaging interval for the filter. The averaging constant is usually set as 0.01 or 0.02 for smoothing intervals of 100 or 50 s respectively, for measurements at the rate of 1 Hz. It should be noted that, since this filter uses the time-differencing of the carrier phase measurements, it must be reset whenever a cycle slip occurs to account for changes in the integer ambiguity.

## 3. Results

### 3.1. Experimental Setup

For this paper, two different 24 h surveys were used. The first survey used GPS data from the International GNSS Service (IGS), captured using a geodetic-grade receiver in ESA’s Malargüe Satellite Tracking Station, in Argentina [20,21]. The second survey was conducted at the GNSS Laboratory of Instituto de Telecomunicações, in Lisbon. Data collection for this survey was performed using two different types of receivers: two u-blox 6T receivers from u-blox (Thalwil, Switzerland) with NovAtel GPSAntenna Model 521 antennas from Novatel Inc. (Calgary, Canada) and an Ashtech ProFlex 500 receiver with an AT1675-7M antenna, both from Ashtec, now Magellan (San Dimas, California, U.S.A.). These receivers were connected to a laptop computer which provided both serial communication handling and internet protocol (IP) communication. The resulting surveys were saved in RINEX 3.02 format. The two u-blox receivers were also used for a DGPS test based on the initial results of this paper. In this setup, the antenna designated RF2 worked as base station, while the antenna RF6 worked as a rover (the naming scheme of the antennas is specific for the IT GNSS Laboratory and has no correlation with GNSS marker names). The data collection was performed in clear meteorological conditions to avoid signal degradation and to provide a good performance baseline. It should be noted that these conditions correspond to the expected real-world conditions for the expected uses of this methodology, so there is a good expectation of obtaining comparable results.

For all the positioning methods, the ionospheric and clock modeling used is as defined in [22], and the tropospheric modeling follows that in [23].

### 3.2. Hatch Filter Averaging Constant Determination

To obtain the Hatch Filter averaging constant that best fits the four different methods, four values were tested: 0.005, 0.01, 0.015, and 0.02. The results are presented in Table 1. It was observed that a higher value improves the LS and WLS mean error, at the cost of a slightly decreased precision, but only at the decimeter level. For the EKF and UKF, a lower value for the constant yielded better results in all metrics, but in the order of millimeters. A middle-ground value of γ=0.010 was then chosen for the rest of the simulations in order to quantify the impact of the Hatch Filter in the positioning methods.

### 3.3. Base Station Antenna Position Determination

Using the previous positioning methods, the position of antenna RF2, from here on also designated as the base station, was measured for the entire survey. The obtained results are presented in Table 2.

From these results, it is clear that both EKF and UKF present a much more precise and accurate position solution, with an error below the meter level. The Hatch Filter also results in a lower spread for the LS and WLS results but produces a slightly higher mean error, while for the EKF and UKF, the effect is opposite.

### 3.4. Convergence over Time of the Position Solution

**Figure 3:**
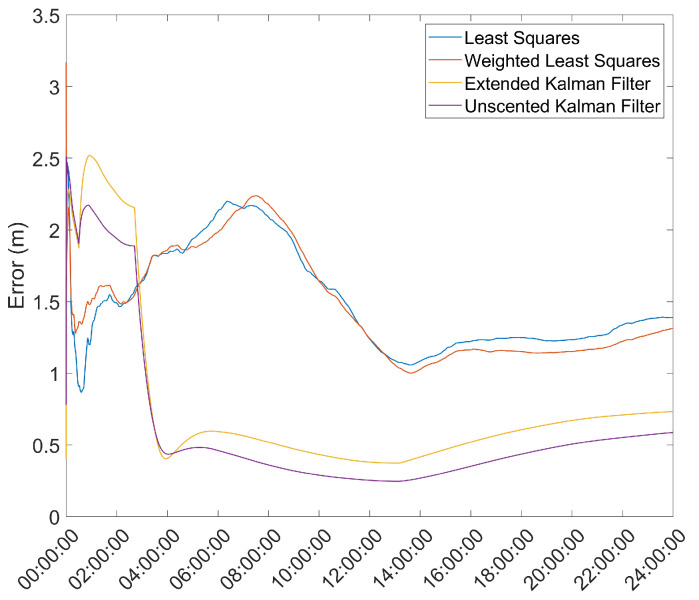
Mean error variation over time for the base station antenna with no Hatch Filter.

**Figure 4:**
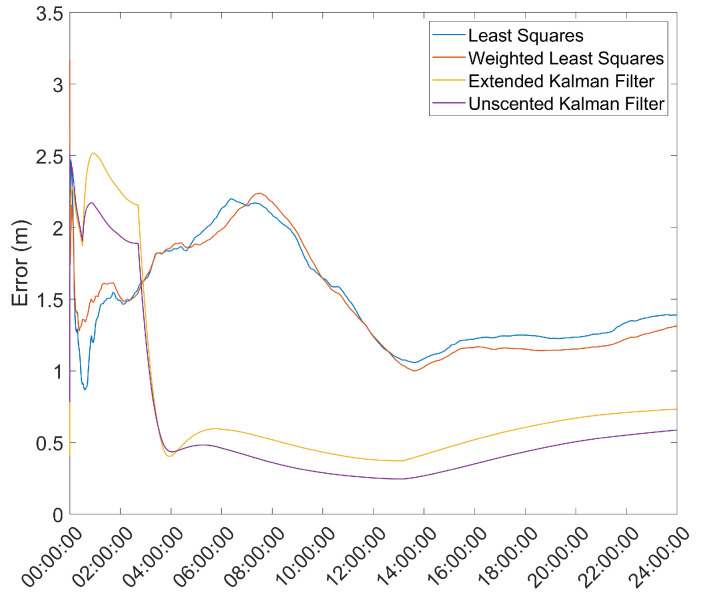
Mean error variation over time for the base station antenna with Hatch Filter and γ=0.010.

Another important metric is the time necessary for the position solution to converge to below a given error threshold. As such, the mean error over time was computed and is presented in Figure 3 and Figure 4.

**Figure 5:**
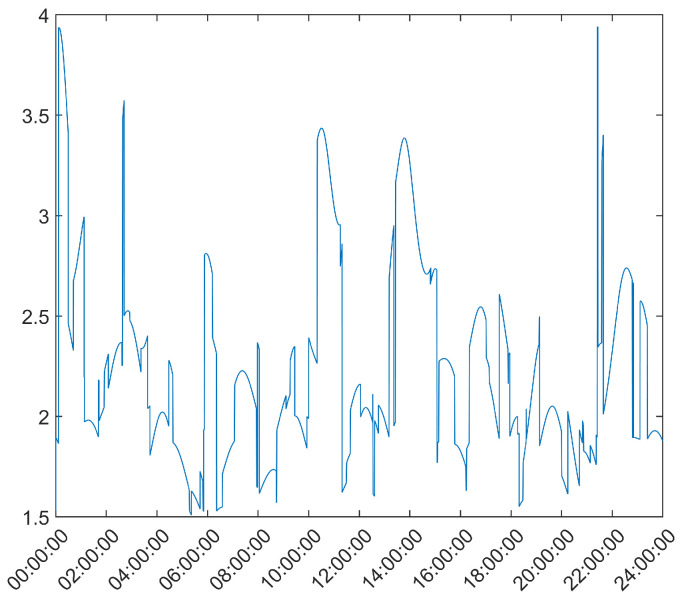
GDOP values for the 24 h survey of the base station antenna.

From here, it is clear that the Hatch Filter does not produce significant changes to either of the mean error dynamics. Furthermore, while the EKF and UKF show a higher initial error, that error falls to values below those of the LS and WLS in only a few hours. From here, a clear trend of more accurate results for the EKF and UKF can be seen, with a lead for the UKF. It should be noted that there is an increase in the mean error after approximately 13 h of the survey; most likely, that is the result of constellation changes, as can be seen from Figure 5, where there is a jump in the GDOP parameter to much higher values.

### 3.5. Position Estimate Filtering Using a Threshold

**Figure 6:**
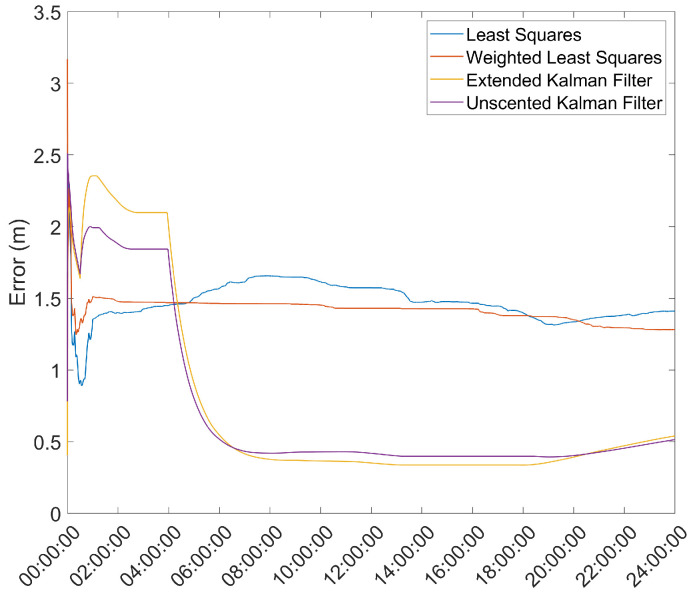
Effect of a 1σ threshold on the mean error over time for the reference station antenna with no Hatch Filter.

**Figure 7:**
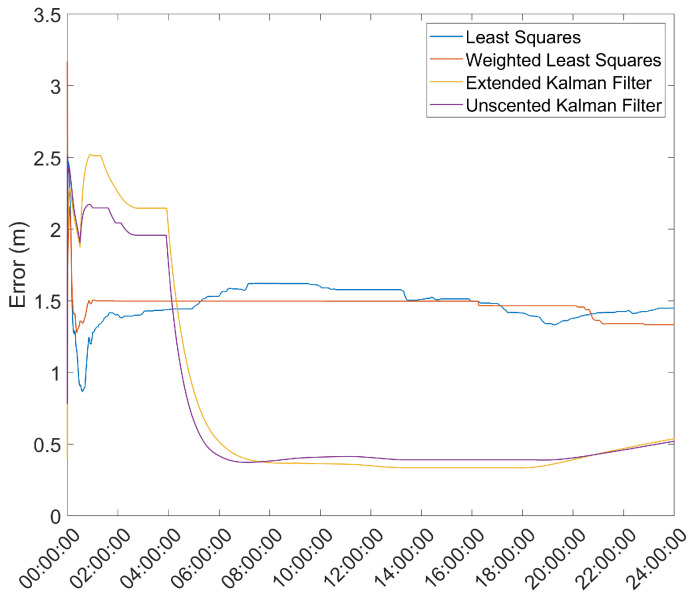
Effect of a 1σ threshold on the mean error over time for the reference station antenna with Hatch Filter and γ=0.010.

**Figure 8:**
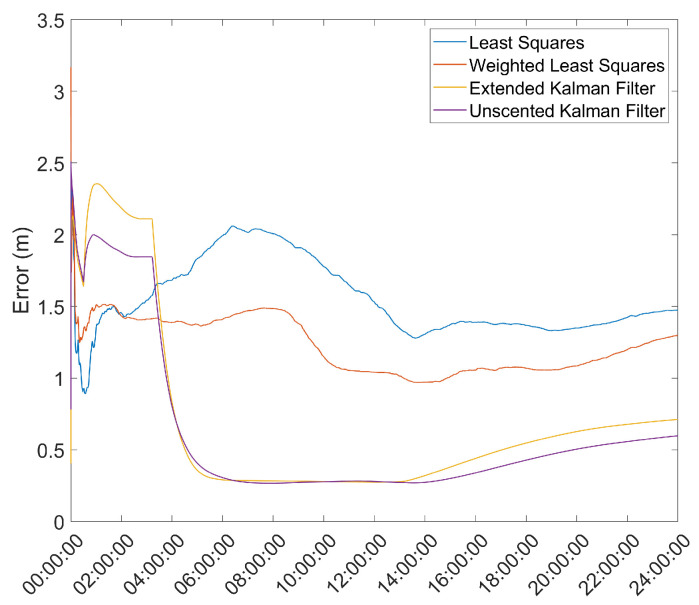
Effect of a 2σ threshold on the mean error over time for the reference station antenna with no Hatch Filter.

**Figure 9:**
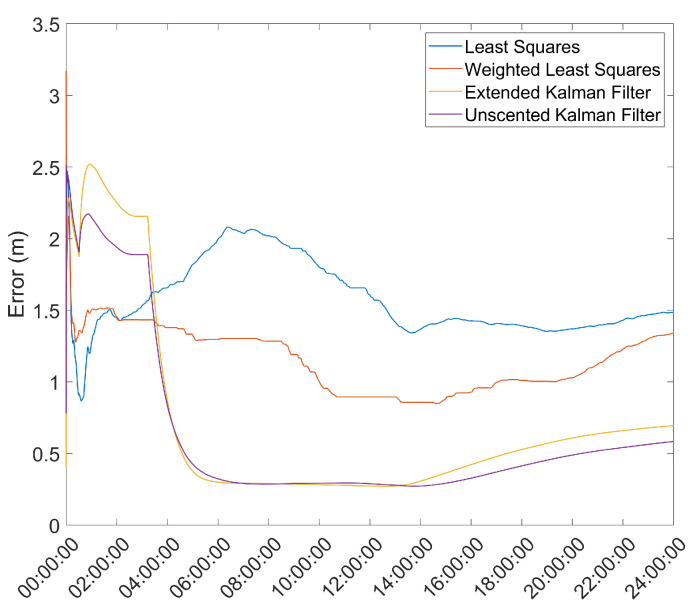
Effect of a 2σ threshold on the mean error over time for the reference station antenna with Hatch Filter and γ=0.010.

To improve the mean position estimate, a threshold filter based on the standard deviation, σ, of the position solutions was used, with two different thresholds: 1σ and 2σ. This filter works by taking the mean and standard deviation of the position estimates during the first hour and then uses those parameters to filter outliers in the data set, continuously updating the mean and standard deviation after the first hour. The results are presented in Figure 6, Figure 7, Figure 8 and Figure 9.

From these results, it is seen that for the 2σ threshold, the EKF and UKF present a lower mean error but allow greater variation in the position solutions; meanwhile, for the LS and WLS, this greater variation allows an increase in the estimate error. For the 1σ threshold, the results show significantly smaller variations for all methods but an increase in the mean error.

### 3.6. Final Proposed Positioning Method

**Figure 10:**
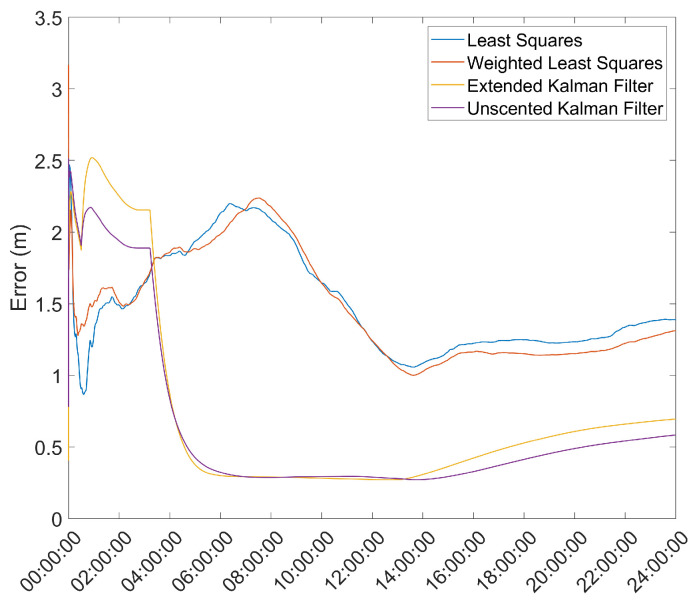
Mean error for 24 h position survey of the reference station antenna using the finalized position methods.

In order to obtain a final positioning method, the combination from the previous results that yielded the best overall performance was chosen. For the LS and WLS, this corresponds to the case without threshold filtering and with a Hatch Filter with γ=0.010; In the case of the EKF and UKF, it is the case with a 2σ threshold and a Hatch Filter with γ=0.010. The error metrics in these conditions are presented in Table 3 and the mean error for the whole survey is given in Figure 10.

While using a Hatch Filter for the LS and WLS increases the mean error slightly, it also reduces the noise in the position estimates significantly, allowing for much more precise, even if slightly less accurate, position estimates; for this reason, it was opted to be included in the final proposed method.

### 3.7. Performance Comparison with Existing Auto-Survey Methods

**Figure 11:**
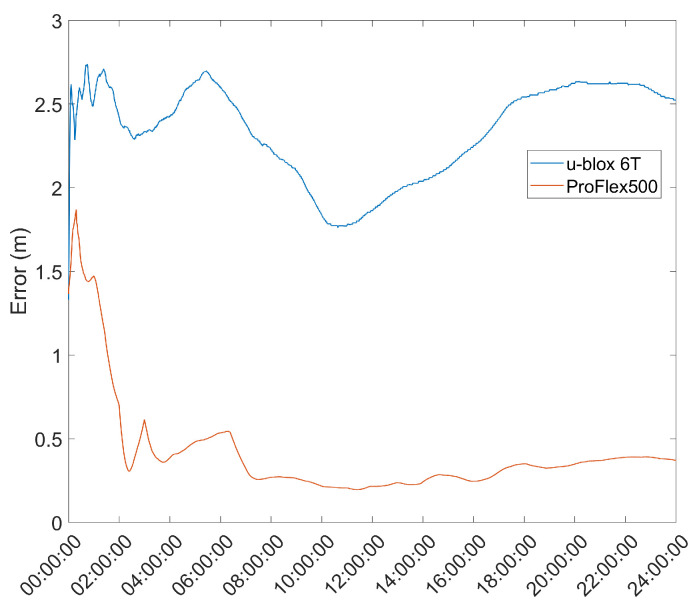
Auto-survey results for the u-blox 6T and ProFlex receivers for a 24 h survey.

In order to benchmark the results of the proposed methods, they were compared against known auto-surveying methods. To this end, the survey-in mode of the u-blox 6T receivers was used, as well as the static EKF positioning method of RTKLIB [24] using the ProFlex 500 as a receiver. These surveys were done in the same conditions and for the same time, and the results are presented in Figure 11.

Comparing these results with the results presented in Figure 3 and Figure 4, it can be seen that the EKF and UKF methods proposed provide a significantly better estimate than the survey-in mode, and, although worse, within 25 cm, after roughly 4 h, of the results obtained with the ProFlex 500 receiver, which is a much higher quality receiver than the u-blox 6T used in the survey.

### 3.8. DGPS Test

**Figure 12:**
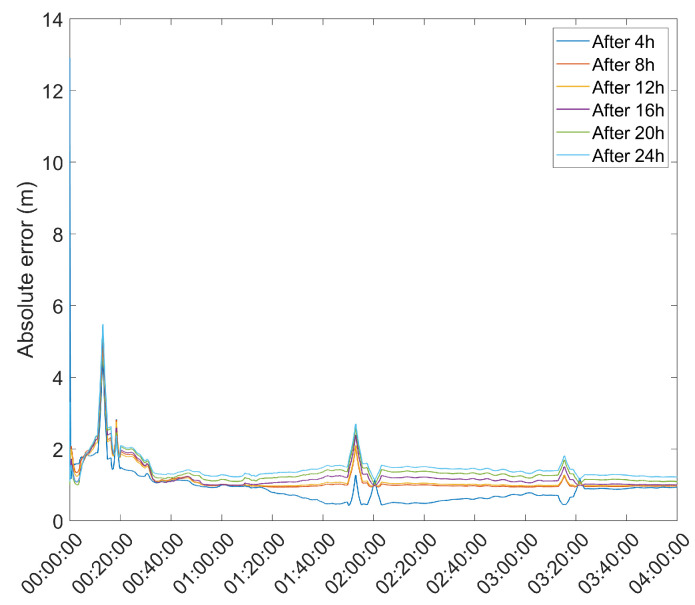
Rover position error with base station position obtained with EKF and averaged after a set number of hours.

**Figure 13:**
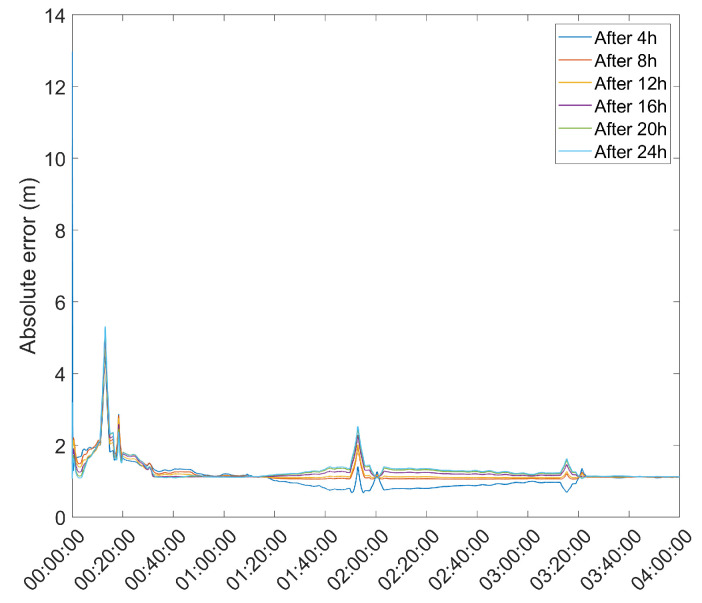
Rover position error with base station position obtained with UKF and averaged after a set number of hours.

As a final test of the proposed methods, a DGPS test using a Double Difference DGPS setup as defined in [2] was performed. The choice of DGPS for the test of the proposed methods, instead of RTK, stems from the fact that, in the present paper, only pseudorange-based positioning was performed, which makes DGPS the most suitable method to benchmark the results of the present research. This test used the u-blox 6T receiver connected to an RF2 antenna from the previous tests as a base station, and another, identical receiver–antenna pair as a rover, with the antenna designated as RF6. This test took the mean position estimate of the base station since the start of the survey at 4 h intervals, using both EKF and UKF to estimate the base station position, and used that estimate to determine the rover receiver during a 4 h survey. The resulting mean error is presented in Table 4, with the rover position error during the survey presented in Figure 12 and Figure 13.

## 4. Discussion

To characterize the performance of several methods for the auto-survey procedure of a GNSS base station, the LS, WLS, EKF, and UKF methods were tested using data from a 24 h survey. To enhance the results of these methods, both a carrier-smoothed code method, the Hatch Filter, and a threshold filter were also analyzed.

The analysis of these results shows a very clear benefit of the EKF and UKF compared to the LS and WLS, with the UKF showing slightly better results than the EKF. From these results, the LS and WLS do not appear to be viable auto-survey methods; hence, the EKF and UKF should be used for this purpose. In addition, while the UKF shows a slightly better result than the EKF, it also has higher computational complexity due to the sampling of the sigma points and their propagation through the non-linear function.

Comparisons with existing auto-survey methods show that the EKF and UKF provide significantly better results when compared to the survey-in algorithm of the u-blox 6T receiver but show a worse estimate than that of the ProFlex 500 receiver; this is to be expected, since the u-blox 6T receiver used is an low-cost, single-frequency receiver when compared to the geodetic grade, dual-frequency ProFlex 500. It should be noted, however. that the EKF and UKF mean position estimate is still very close to that of the much more expensive ProFlex 500 receiver, with both having decimeter-level accuracy.

When applied to a DGPS setup, the good performance of the EKF and UKF methods for auto-survey can be seen, with the test showing estimates with error of about 1 m or less for the rover position for all auto-surveys using the UKF and slightly worse results for the EKF. These results are promising, and further research could improve them, allowing for even greater precision.

Another important conclusion is that there is a precision threshold at roughly the 4 h mark, with only marginal improvements for longer surveys. This result is important for the stated applications of the presented methods, as it can be used, together with the estimate precision data, to help the intended audience of this paper guide planning and logistics for said activities to better account for the auto-survey procedure without requiring an arbitrarily long survey time.

These results show that low-cost GPS base stations are achievable with inexpensive receivers and small survey windows, without requiring extensive surveys, high-end survey equipment, or other external inputs and still provide meter-level or below accuracy for rovers. This opens the possibility of creating very low-cost GPS base stations for differential positioning methods without requiring external augmentations, paving the way for the use of precise GPS position estimation in activities that cannot justify the added cost of high-precision receivers and in remote and GNSS-underserved areas at a fraction of the cost of a commercial high-end receiver.

## Figures and Tables

**Table 1:** Error metrics for different values of γ of the Hatch Filter for the ESA Malargüe receiver.

	LS				WLS				EKF				UKF			
γ	**0.005**	**0.010**	**0.015**	**0.020**	**0.005**	**0.010**	**0.015**	**0.020**	**0.005**	**0.010**	**0.015**	**0.020**	**0.005**	**0.010**	**0.015**	**0.020**
Mean (m)	1.342	1.333	1.331	1.331	1.109	1.084	1.077	1.073	0.983	0.984	0.984	0.985	0.983	0.984	0.984	0.985
RMS (m)	3.685	3.669	3.665	3.663	3.295	3.264	3.255	3.251	1.335	1.340	1.342	1.342	1.334	1.340	1.341	1.342
DRMS (m)	2.112	2.119	2.124	2.126	1.858	1.859	1.861	1.862	0.634	0.638	0.640	0.641	0.634	0.637	0.640	0.641
MRSE (m)	3.432	3.419	3.414	3.413	3.103	3.078	3.071	3.068	0.902	0.910	0.912	0.912	0.902	0.909	0.912	0.912

**Table 2:** Error metrics for 24 h position survey of the base station antenna.

	No Hatch Filter				Hatch Filter with γ=0.01			
**Error Metric**	**LS**	**WLS**	**EKF**	**UKF**	**LS**	**WLS**	**EKF**	**UKF**
Mean (m)	1.386	1.300	0.750	0.602	1.388	1.312	0.732	0.587
DRMS (m)	1.589	1.557	0.818	0.750	1.341	1.377	0.826	0.755
MRSE (m)	2.264	2.249	1.558	1.349	1.990	2.028	1.565	1.355

**Table 3:** Error metrics for 24 h position survey of the reference station antenna using the finalized position methods.

Error Metric	LS	WLS	EKF	UKF
Mean (m)	1.386	1.300	0.694	0.584
RMS (m)	2.655	2.597	1.536	1.314
DRMS (m)	1.589	1.557	0.740	0.676
MRSE (m)	2.264	2.249	1.370	1.177

**Table 4:** Mean error for the DGPS receiver with base station position averaged after a set number of hours.

	Extended Kalman Filter						Unscented Kalman Filter					
**Hours**	**4**	**8**	**12**	**16**	**20**	**24**	**4**	**8**	**12**	**16**	**20**	**24**
Mean error (m)	0.732	0.794	0.661	0.787	0.903	0.932	0.767	0.677	0.506	0.584	0.686	0.719

## Data Availability

The IGS observation data used in this paper can be found at ftp://gssc.esa.int/gnss/data/highrate/2020/299/ (accessed on 2 November 2020) and the navigation data at ftp://gssc.esa.int/gnss/data/hourly/2020/299 (accessed on 2 November 2020), in the files from GNSS marker MGUE. The captured survey data from the GNSS Laboratory of Instituto de Telecomunicações are openly available at DOI:10.5281/zenodo.5524997 (accessed on 23 September 2021).
